# Inference of germinal center evolutionary dynamics via simulation-based deep learning

**Published:** 2025-08-13

**Authors:** Duncan K Ralph, Athanasios G Bakis, Jared Galloway, Ashni A Vora, Tatsuya Araki, Gabriel D Victora, Yun S Song, William S DeWitt, Frederick A Matsen

**Affiliations:** 1 Fred Hutchinson Cancer Research Center, Seattle, Washington, USA; 2 Department of Statistics, University of California, Irvine, CA; 3 Department of Genome Sciences, University of Washington, Seattle, WA; 4 Laboratory of Lymphocyte Dynamics, The Rockefeller University, New York, NY; 5 Department of Statistics, University of California, Berkeley, CA; 6 Computer Science Division, University of California, Berkeley, CA; 7 Department of Statistics, University of Washington, Seattle, WA; 8 Howard Hughes Medical Institute, Seattle, WA

## Abstract

B cells and the antibodies they produce are vital to health and survival, motivating research on the details of the mutational and evolutionary processes in the germinal centers (GCs) from which mature B cells arise. It is known that B cells with higher affinity for their cognate antigen (Ag) will, on average, tend to have more offspring. However the exact form of this relationship between affinity and fecundity, which we call the “affinity-fitness response function”, is not known. Here we use deep learning and simulation-based inference to learn this function from a unique experiment that replays a particular combination of GC conditions many times. All code is freely available at https://github.com/matsengrp/gcdyn, while datasets and inference results can be found at https://doi.org/10.5281/zenodo.15022130.

## Introduction

The germinal center (GC) is the site of affinity maturation of B cell receptors (BCRs), and as such is of central importance to the functioning of the adaptive immune system. Naive B cells, after encountering their cognate antigen, migrate to GCs, where they alternate cycles of proliferation and mutation (in the GC’s dark zone) and affinity-based selection (in the light zone) (Cyster and Allen, 2019; Victora and Nussenzweig, 2022). Over time scales of several weeks (and many generations) this results in an increase in the average affinity of the population of B cells (Berek and Milstein, 1987; Allen et al., 1988). Understanding these processes that occur in the GC is thus of great importance both to achieving a fundamental grasp of the immune system, and to advancing goals such as making a vaccine for difficult-to-neutralize viruses (Burton et al., 2012).

The GC is a Darwinian evolutionary system designed to improve affinity, and as such one might ask: what relationship between affinity and fitness does it use? In order for the GC reaction to improve affinity, this relationship must be increasing. That is, on average, B cells with higher affinity BCRs will reproduce more than those with low affinity BCRs, leading to an overall improvement in affinity. However, the details of the relationship between affinity and fitness are unknown. This is in part because the fitness of a B cell is an emergent property of an intact, functioning germinal center and thus cannot be measured using an in vitro assay.

There have been a variety of suggestions for the form of the affinity-fitness relationship (Batista and Neuberger, 1998; Kuraoka et al., 2016; Murugan et al., 2018). Resolving these possibilities in practice, however, requires care, even in the ideal situation of single-cell sequencing of individual germinal centers (Tas et al., 2016; DeWitt et al., 2025). One cannot simply examine by hand trees built from observed sequences and gain insight. Indeed, processes such as the “push of the past” and the “pull of the present” (Nee et al., 1994; Budd and Mann, 2018) can mislead interpretation of germinal center sequence data. Instead, an approach is needed that can model extinct lineages that do not appear in the reconstructed tree (DeWitt et al., 2025).

We will approach the affinity-fitness relationship via a birth-death model in which the birth rate is a function of affinity; we call this function the “affinity-fitness response function.” We assume a family of such functions, determined by some set of parameters, such that a choice of parameters gives a specific response function. We will use data from the “replay” experiment (DeWitt et al., 2025) to fit this function. This experiment used mice whose GCs are seeded entirely by B cells with a single, fixed naive antibody. They were immunized with this naive antibody’s cognate antigen, and the subsequent GC reactions were observed in two ways: by extracting and sequencing individual GCs near a single timepoint, and by pooling multiple GCs at each of a wide range of timepoints for single-cell sequencing. In addition, a deep mutational scan (DMS) on the naive antibody’s sequence was performed for binding to the immunogen. This DMS enables us to make predictions about the affinity of an arbitrary sequence. These affinities can then be input into the affinity-fitness response function; one can compare the observed phylogenetic trees to predictions made by the corresponding birth-death process to learn about the parameters of the response function.

Ideally one would be able to fit the parameters of the affinity-fitness response function via maximum likelihood or Bayesian inference. However, this is difficult. In general, calculating likelihoods for birth-death models is a challenge, requiring solutions of ordinary differential equations (ODEs) that take the probability of unsampled lineages into account. Bakis et al. (2025) have employed this approach, using the multitype birth-death (MTBD) model of (Kühnert et al., 2016; Barido-Sottani et al., 2020) but adapting it to the case of many trees evaluated in parallel. The types in the MTBD are discretized bins of affinity, and mutation moves lineages between bins.

This approach has the substantial advantage of being rigorously tractable using numerical integration of ODEs and Markov chain Monte Carlo (MCMC); however, the inferential model is misspecified in two ways. First, it requires Markov evolution of the types in the MTBD model. In our case, and described in more detail below, the type is determined by the sequence, and so the type evolution is determined by sequence evolution. Because the relationship between sequence and affinity type is non-trivial, and the sequence-based mutation process is complex, the evolution of the types is not Markovian. Second, the MTBD model cannot accommodate population size constraints. Models with such constraints in fact generally have intractable likelihoods because the evolution of each cell depends on the entire population of cells. Real germinal centers are subject to such population constraints, reaching them around 10 days, well before our sampling for these experiments at 15 or 20 days (Schwickert et al., 2007; Mesin et al., 2020).

Likelihood-free inference is an alternative approach to fitting complex statistical models. Classical likelihood-free inference uses Approximate Bayesian Computation with summary statistics (Beaumont et al., 2002). More recently, techniques using deep neural networks have emerged, especially in population genetics (Sheehan and Song, 2016; Schrider and Kern, 2018). Recent work has extended this to the inference of phylogenetic model parameters (Voznica et al., 2022; Lajaaiti et al., 2023; Landis and Thompson, 2025) including state-dependent diversification models (Lambert et al., 2023; Thompson et al., 2024). Can we use these recent methods to learn the parameters of the affinity-fitness function in the germinal center?

**Figure 1.**
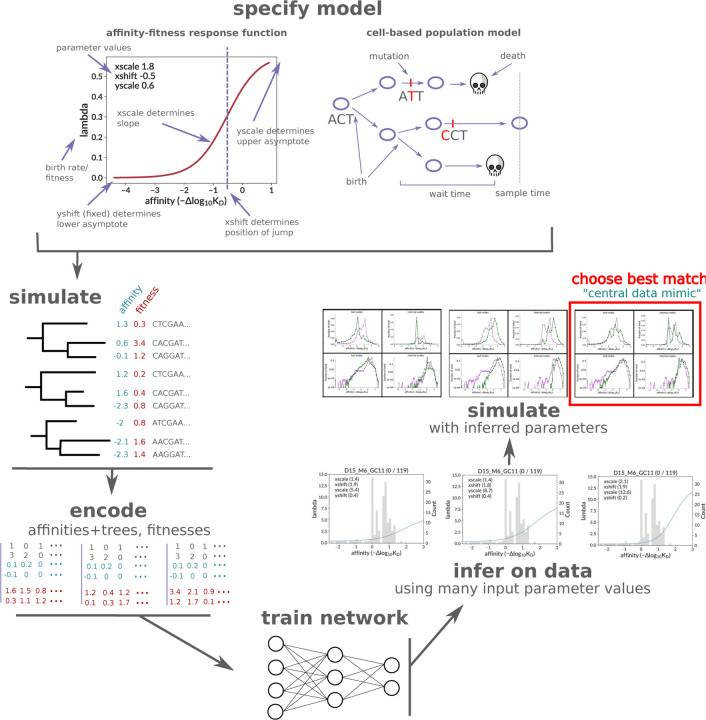
Overview of the workflow for this paper. After specifying a birth-death model with sigmoid affinity-fitness response function, we simulate many trees and their sequences at each node, with parameters roughly consistent with our real data samples. The simulation is cell-based and implements a carrying-capacity population size limit. The results of the simulation are then encoded and used to train a neural network that infers the sigmoid response parameters on real data. In addition to encoded trees, the network also takes as input the assumed values of several non-sigmoid parameters (carrying capacity, initial population size, and death rate). Inference on real data is performed many times, with many different combinations of non-sigmoid parameter values, and additional “data mimic” simulation is generated using each of the resulting inferred parameter value combinations. The summary statistics of each data mimic sample are compared to real data, with the best match selected as the “central data mimic” sample with final parameter values for both sigmoid (inferred with the neural network) and non-sigmoid (inferred by matching summary statistics) parameters. Figure 1—figure supplement 1. Simulation response function example with sampled affinity values. Figure 1—figure supplement 2. Diagram of curve difference loss function calculation. Figure 1—figure supplement 3. Example of approximate sigmoid parameter degeneracy.

In this paper, we learn the relationship between affinity and fitness via simulation-based inference using cell-based forward simulation, neural network inference, and summary statistics matching (Figure 1). We begin from the compact bijective ladderized vector (CBLV) tree encoding of Voznica et al. (2022), adding affinities for each node from ancestral sequence reconstruction and the sequence-affinity mapping. Our forward model contains too many parameters to be inferred using neural networks alone, so we complement the neural network inference with summary statistic matching for some parameters. There are two quantities in our model that can be described as fitnesses or birth rates: an intrinsic rate (the response function above), which is fixed for a given parameter set and which describes a cell’s birth rate in the absence of capacity constraints; and the effective (net) rate, which incorporates these constraints while subtracting the death rate, and varies with time and GC properties. We infer response functions on 119 trees from (DeWitt et al., 2025), finding them to be generally similar across GCs, and roughly tripling from affinity 0 (naive) to 1, and then tripling again from 1 to 2. This means that a cell that mutated to affinity 1 in the early stages of the GC would replicate roughly three times as fast as the cells around it. The effective, or net, birth rate lies within much narrower bounds once the GC nears carrying capacity, between roughly −0.2 and 1 for most GCs.

## Methods

### Overview of the replay experiment and data

In order to quantify the mechanics of GC selection we use data from an experimental mouse system that we nickname the “replay” experiment (DeWitt et al., 2025) in which all naive B cells seeding GCs are identical, carrying the same pre-rearranged IG genes. Thus, the starting sequences for affinity maturation in these GCs have identical affinity and specificity for their cognate antigen. This antigen is chicken IgY, which has been characterized previously (Tas et al., 2016; Jacobsen et al., 2018). The naive sequence chosen (called “clone 2.1”) was the naive version of a dominant sequence in a GC with a very large clonal burst 10 days after immunization (Tas et al., 2016), so we expect that it is a good starting point for affinity maturation. The engineered mice carry the unmutated versions of the Igh and Igk genes rearranged by clone 2.1 in their respective loci. They were immunized with chicken IgY, and the resulting GC reactions were observed in two ways, resulting in two separate data sets. For the first, which we refer to as the “extracted GC data”, 119 individual GCs were extracted and sequenced from 12 such mice at either 15 days (52 GCs) or 20 days (67 GCs) after immunization. In the second, called “bulk data”, multiple GCs from the whole lymph nodes of several mice were pooled together at each of seven time points (from 5 to 70 days after immunization), and then analyzed with droplet-based sequencing. In this paper, we only use the extracted GC data; however we also compare our results to those derived from the bulk data.

We infer trees with IQ-TREE (Minh et al., 2020) version 1.6.12 using all observed BCR sequences from each GC with the known naive sequence as outgroup, and ancestral sequence reconstruction by empirical Bayes. We use the branch lengths from IQ-TREE directly, without attempting to infer calendar times for the internal nodes.

These BCR trees are annotated with antibody affinities at all nodes as follows. First, we perform ancestral sequence reconstruction for every node of the phylogenetic tree. Because these trees have relatively few mutations, this can be done with low error. We first take affinity values for each single-mutation variant from a DMS experiment (DeWitt et al., 2025) that used methods similar to Adams et al. (2016). For sequences with more than one mutation, we simply add the effect of each individual mutation (i.e. we assume no epistasis), which was shown to be a reasonable approximation in DeWitt et al. (2025).

The 119 trees have between 26 and 95 leaves, with a mean (median) of 74 (78) leaves. Observed sequences have between 0 and 19 nucleotide mutations, with a mean (median) of 6.3 (6.0) nucleotide mutations. Affinity (defined below) is 0 for the initial unmutated sequence, and ranges from −37 to 3 in observed sequences, with a mean (median) of −0.3 (0.3).

### Model

In order to simulate sequences and trees that we will then use to train the neural network, we employ a birth-death model with a population size constraint. Our model is designed to mimic the replay experiment, with a parameterized affinity-fitness response function mapping each cell’s affinity to its fitness. We provide more detail below and in Table 1, but to summarize, we make the following assumptions about the birth-death-mutation process driving the germinal center:

- The GC is seeded with an initial population size and has a fixed upper limit (carrying capacity) on the GC population size.
- Birth events happen with a per-cell rate given by the response function, which is then logistically modulated as the population nears carrying capacity.
- Death events happen at one of two constant rates, one for cells with functional sequences, the other for nonfunctional ones (e.g. with stop codons).
- Mutation events happen on the edges of the developing phylogenetic tree according to a process that depends only on the mutability of the evolving sequence using a context-sensitive model (Cui et al., 2016).
- The process is stopped after a fixed period of time, and a sample of a given size is taken uniformly.
- If the number of living cells falls to zero, or if there are fewer than 10 living cells at sampling time, the tree is discarded and the simulation is retried.

The simulation is initialized with a single root node with the naive sequence from the replay experiment. This initial node then immediately undergoes repeated binary expansion with neither mutation nor time passage until we have the desired number of initial naive sequences.

Each subsequent step in the simulation process consists of choosing one of the three possible events as follows. We loop over all event types and all nodes, calculating a waiting time for each such node-event type combination. These waiting times are calculated from an event type-specific response function, which relates each node’s affinity to the likelihood, or rate, of that event type. For instance the birth response function mentioned above relates the node’s affinity to the rate at which it divides (i.e. at which birth occurs). The waiting time for each node-event type combination is sampled from the inverted response function (which, as an event rate, has an inverse of time). For mutation events, the waiting time distributions are calculated using the SHM model mutabilities summed over each node’s sequence. The event type-node combination with the smallest waiting time is then selected to occur, and the corresponding cell is removed from the population. If a birth event is selected, two identical children are added to the population. For a death event, no cells are added. For a mutation, the location of the substitution and the new base are chosen according to probabilities from 5-mer sequence contexts in the node’s sequence using the model of Cui et al. (2016); a single new cell is created to replace the old.

Inspired by Dessalles et al. (2018), we enforce the germinal center’s carrying capacity by logistically modulating the birth rate such that the process is critical (average birth and death rates over living cells are equal) when the population is at carrying capacity. We modulate each cell’s birth rate by applying a multiplicative factor m:

(1) $$m={\left(\frac{\sum_{i}\mu_{i}}{\sum_{i}\lambda_{i}}\right)}^{N\text{/}N_{0}}$$

which, for carrying capacity $N_{0}$, sums death rates $\mu_{i}$ and birth rates $\lambda_{i}$ over the N living cells. For initially small populations, the exponent is near zero and no modulation occurs. As the population increases, however, the exponent goes to one. Thus, as evolution increases affinities and, consequently, birth rates (in the denominator), m becomes small, decreasing each $\lambda_{i}$. Thus initial exponential growth (a supercritical process) gradually slows as it approaches criticality at the specified capacity. If the population fluctuates above carrying capacity, the exponent $N\text{/}N_{0}>1$ causes m to decrease rapidly, which in turn decreases the birth rates in its denominator. We have also experimented with two other methods of carrying capacity enforcement, but which were not used for any results in this paper. They are nevertheless important to mention, since we do not know which method best reflects real GC dynamics, and here simply assume birth modulation. One method modulates death, rather than birth, using:

(2) $$m={\left(\frac{\sum_{i}\lambda_{i}}{\sum_{i}\mu_{i}}\right)}^{N\text{/}N_{0}}.$$

While the other, called “hard”, immediately kills a random individual whenever a birth event causes the population to exceed the carrying capacity.

A variety of birth response functions (relating a node’s affinity to its birth rate) are implemented, but all simulations in this paper use a sigmoid on affinity x (Figure 1—figure Supplement 1):

(3) $$\lambda \left(x\right)=\frac{y_{c}}{1+e^{-x_{c}\left(x-x_{h}\right)}}+y_{h}$$

with affinity defined relative to the naive dissociation constant ($K_{D}^{N}≃40nM=4x\;10^{-8}M$ (Tas et al., 2016)):

(4) $$x=-\log_{10}K_{D}\text{/}K_{D}^{N}$$

such that, for example, an affinity of +2 (−2) means a $K_{D}\;0.01\left(100\right)$ times the naive value. Recall that lower $K_{D}$ means stronger binding, so larger x corresponds to stronger binding. In the real data, we observe affinities from approximately −37 to 3. The sigmoid’s upper asymptote $\left(y_{h}+y_{c}\right)$ represents a hypothesized fitness ceiling above which further affinity increases do not improve fitness. Its lower asymptote $\left(y_{h}\right)$ gives the fitness of very low-affinity nodes (due, for instance, to tonic signaling), which represent cells with either nonfunctional or severely compromised receptors. The x position of the transition between these asymptotes is given by $x_{h}$, while $x_{c}$ determines the transition’s steepness. We use the following more descriptive names when plotting: xshift means $x_{h}$, xscale means $x_{c}$, yscale means $y_{c}$, and yshift means $y_{h}$, but keep the more compact names for the rest of this section.

To construct our training sample, we thus want to sample $x_{c}$, $x_{h}\;y_{c}$, and $y_{h}$ values from throughout biologically plausible ranges. One cannot, however, choose the value of each parameter independently without yielding unrealistic initial birth rates. Besides poorly describing real GCs, such rates would result in many failed simulations. Along with bounds on each of these parameters, we thus also incorporate bounds on the naive (zero-affinity) birth rate, namely

(5) $$\lambda_{0}=\frac{y_{c}}{1+e^{x_{c}x_{h}}}\text{,}$$

with lower and upper bounds $\lambda_{0}^{l}$ and $\lambda_{0}^{u}$. Note that here we have neglected $y_{h}$, and choose $y_{h}$ within its own bounds, independently of the other parameters. This amounts to the approximation that tonic signaling is small compared to the fitness signal from high affinity $\left(y_{h}<<y_{c}\right)$. We then choose each remaining parameter in an arbitrary, but consistent, order with the following procedure. We first choose a value for $x_{c}$ from within its bounds $\left[x_{c}^{l},x_{c}^{u}\right]$ (Table 1), and substitute that value into the $\lambda_{0}$ constraints. This gives us an additional constraint on the next parameter, $x_{h}$, which also has its own bounds $x_{h}^{l}$ and $x_{h}^{u}$:

(6) $$\frac{\log\left(\frac{y_{c}^{l}}{\lambda_{0}^{u}}-1\right)}{x_{c}}<x_{h}<\frac{\log\left(\frac{y_{c}^{u}}{\lambda_{0}^{l}}-1\right)}{x_{c}}.$$

Analogous additional constraints are then also incorporated when subsequently choosing the last parameter $y_{c}$, with its own bounds $y_{c}^{l}$ and $y_{c}^{u}$:

(7) $$\lambda_{0}^{l}\left(1+e^{x_{c}x_{h}}\right)<y_{c}<\lambda_{0}^{u}\left(1+e^{x_{c}x_{h}}\right)$$

### Tree encoding

We encode each tree with an approach similar to Lambert et al. (2023) and Thompson et al. (2024), enriching the basic CBLV encoding from Voznica et al. (2022) with information on the state of the evolving entity. In contrast to Lambert et al. (2023) and Thompson et al. (2024), however, who labeled only tips with discrete types, we label all nodes with a continuous variable. The variable we are interested in is affinity, which we incorporate into the encoded matrix as two additional rows. Since the original matrix’s two rows represent leaf and internal node heights (Voznica et al., 2022, Fig. 2), each entry in our additional two rows represents the corresponding node’s affinity value (Figure 1).

This tree encoding scheme requires establishing a defined ordering of the nodes such that for each tree, each node has a unique location in the resulting matrix. In the original method (Voznica et al., 2022), however, this correspondence (and thus the encoding) is only unique if all nodes have unique times. For generality, we would like to allow the use of ultrametric trees, with all cells sampled at the same time. This has the potential to confuse the neural network, since it destroys the one-to-one correspondence between trees and encodings. We thus add several tiebreakers to the “ladderization” scheme that establishes the order in which we iterate over nodes. After first sorting nodes by their time, we then also sort any ties by the time of their immediate ancestral node, and then further sort any remaining ties by their affinity. This could, in principle, still result in ties. In practice, however, we have set our code to raise exceptions if ties are encountered, and this has not yet occurred.

For both real and simulated data, we infer trees with IQ-TREE (Minh et al., 2020) (see details above). By taking only sampled sequences and passing them through the same inference method, this treats data and simulation as similarly as possible, which facilitates accurate neural network training and summary statistic comparison. An alternative would be to compare time trees inferred on data, for instance with BEAST (Suchard et al., 2018), to simulation truth trees. Although this is commonly done (Voznica et al., 2022) we reasoned that inference would be more accurate with simulation and data samples on the same footing. Furthermore, our implementation of initial population size creates large numbers of unmutated ancestral sequences near the root of the simulation truth tree, which would have no observable counterpart in data.

As in Voznica et al. (2022), we also scale trees to mean unit depth before encoding in order to facilitate comparison across trees of different depths.

### Neural network architecture, implementation, and training

Our network is detailed in Table 2, but in brief, after input of encoded trees we begin with several convolutional layers interspersed with pooling layers. There follows a series of dense layers with decreasing complexity until we arrive at the final number of outputs. These dense layers take as input, in addition to the output of previous layers, fixed values for several “non-sigmoid parameters” (details below). The input matrices are padded with zeros to a constant, uniform size, despite deriving from trees with different numbers of nodes (in the current study, they are usually padded to 200 columns). We use the exponential linear unit (ELU) activation function for all layers. Our main “sigmoid” network predicts the four sigmoid parameters. However, as a cross check we also train a model that predicts the response function value in discrete bins of affinity, which we call the “per-bin” model. The per-bin model introduces some amount of model independence, how-ever because it is still trained on sigmoid simulation, it has only a limited ability to infer shapes that differ significantly from a sigmoid.

As detailed below, in making our training samples we endeavor to cover the entire space of plausible parameter values, which includes choosing bounds on each of the three sigmoid parameters (Table 1). We find that leaving the inferred parameters entirely unconstrained tends to confuse the neural network, so we impose a “clip function” with bounds on each parameter during network training. We find that for decent performance, these clipping bounds must be somewhat wider than the simulation bounds; but, heuristically, not overly wide (Table 3).

We scale all input variables (branch lengths, affinities, and non-sigmoid parameters) individually to mean 0 and variance 1 over all trees. This scaling is then reversed after prediction. We experimented with similarly scaling output parameters, but this did not improve performance.

We use an Adam optimizer with a learning rate of 0.01, an exponential moving average (EMA) momentum of 0.99, and a batch size of 32, values which were arrived at by performing hyperparameter optimization to minimize loss values. We do not use dropout regularization, since in our tests it resulted in worse performance than using a validation sample to measure overfitting. When training, we use 20% of each sample for testing, and also reserve 10% of the remaining 80% as a validation sample. We train for 35 epochs, which for both sigmoid and per-bin networks was the point at which validation sample performance began to decrease. While during development we trained on a large variety of simulation samples of different sizes and parameter ranges, the results presented in this paper center on a sample of 50,000 trees with the ranges specified in Table 1. We also used samples as large as 100,000 trees, and with substantially more complex networks, but neither change resulted in significant performance improvements. Also note that these optimization and testing steps were performed only on simulation; we ran data inference only once our methods were essentially finalized.

All code for this paper can be found at https://github.com/matsengrp/gcdyn, and all inputs and outputs, along with instructions for running, can be found at https://doi.org/10.5281/zenodo.15022130.

### Curve difference loss function

For a loss function we use a scaled version of the $L^{1}$ distance between the true and inferred functions (Figure 1—figure Supplement 2). This loss, which we refer to as a “curve difference”, is calculated by dividing the area between the curves by the area under the true curve within domain (affinity bounds) [−2.5, 3]. The per-bin model uses the same loss function, but with a coarser discretization (one value for each bin).

An alternate approach would have been to use mean squared error on the inferred parameters, namely the squared difference between inferred and true values over all predictions. In our case, however, this is a poor choice because we care about the sigmoid’s shape, but not about the underlying sigmoid parameter values, which have little individual biological meaning. The curve loss and the mean squared loss on inferred parameters can be quite different: significant simultaneous changes to two parameters can counteract each other, resulting in very similar curve shapes (figure Supplement 3).

### Non-sigmoid parameter inference with summary statistics

In addition to the four sigmoid parameters, which we infer directly, there are other parameters in Table 1 about which we have incomplete information. For some of these, such as the carrying capacity method and the choice of sigmoid for the response function, we simply make a choice and live with the assumption. For most, however, we choose values based on experiment, whether the replay experiment or values from the literature. These values carry significant uncertainty, however, partly from inherent experimental uncertainty, but also because they may represent different biological quantities to those in simulation. For instance, an experimental measurement of the number of B cells in a germinal center might appear to correspond closely to simulation carrying capacity. However if germinal centers are not well mixed, such that competition occurs only among nearby cells, the “effective” carrying capacity that each cell experiences could be much smaller.

Fortunately, in addition to the neural network inference of sigmoid parameters, we have another source of information that we can use to infer non-sigmoid parameters: summary statistic distributions. Since these distributions are in truth affected by all simulation parameters, the best solution would likely be to infer all uncertain parameters with the neural network. This, however, would require passing much more information into a vastly more complicated network (the current tree encoding, for instance, has no information on sequence abundance) that would have to be trained on simulations covering additional dimensions. We thus deemed this approach infeasible for the current paper.

We instead adopt a two-step procedure, inspired by the concept of conditional generation. Recall that our network takes non-sigmoid parameters as input, i.e. it infers sigmoid values contingent on a set of non-sigmoid values. We thus first infer sigmoid parameters on data for many different values of the non-sigmoid parameters. We then take each resulting set of (inferred) sigmoid and (supplied) non-sigmoid values, generate a new simulation sample with each, and compare their summary statistics to data. The sample whose summary statistics most closely match data is interpreted as having the best inferred values for both sigmoid and non-sigmoid parameters. While it would probably be optimal to include all non-sigmoid parameters in this two-step procedure, the combinatorics of scanning over many different variables are prohibitive. We thus only use three non-sigmoid parameters: carrying capacity, initial population, and death rate. These were chosen because their values likely include substantial uncertainty (see above), and because they exert significant control over summary statistic distributions.

### Central (medoid) curve prediction

While our network predicts each sigmoid parameter individually, it is generally only useful to view the resulting curve as a whole. This is because many different sets of sigmoid parameter values can give quite similar shapes, since a change in one parameter can to some extent be compensated by modifying others (Figure 1—figure Supplement 3). Because in practice we learn the shape of the sigmoid only within some uncertainty, we must thus compare predictions using curves, rather than individual parameters. However, because we assume that all GCs have the same response function, we want to choose a central, representative curve encompassing all four predicted parameters at once. We do this by calculating the medoid curve among predictions, with distance our curve difference loss function. We thus calculate, for the predicted curve for each GC, the sum of squared differences to all other predicted curves in the affinity bounds [−2.5, 3]. This domain was chosen because below −2.5 most curves are very similar, while above 3 they diverge dramatically in a region in which we have very few measured affinity values. We then choose the curve with the smallest such sum as the medoid curve.

## Results

### Deep learning effectively infers parameters on simulation

**Figure 2.**
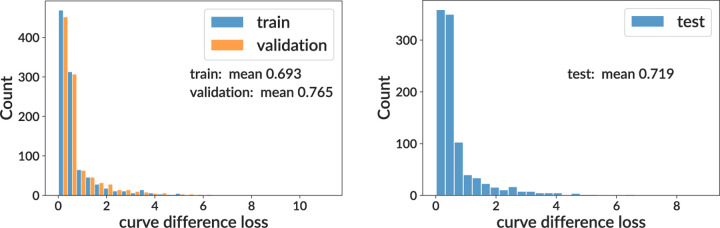
Training and testing results for the sigmoid model on simulation. We show curve difference loss distributions on several subsets of the training sample (where each GC has different parameter values): training and validation (left) and testing (right). For computational efficiency when plotting, the curve difference distributions display only the first 1000 values. See Figure 3 for per-bin model.

**Figure 3.**
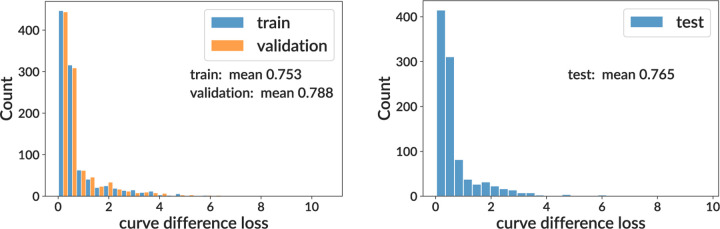
Training and testing results for the per-bin model on simulation. See Figure 2 for details.

After building and training our neural network, we evaluate its performance on training and testing samples. We show values of the curve difference loss function on the training sample (split into training, validation, and test subsets) for both sigmoid and per-bin models (top rows of Figure 2 and Figure 3). This shows that for both models, the mean area difference on a single GC between true and inferred curves is around 70% of the full plot area. We find that the sigmoid and per-bin models have similar overall performance. While this is noisy, these inferences are only for a single GC’s worth of data, whereas for inference on real data we have 119 GCs with the same parameters. Below we show a validation more concordant with the type of analysis done on real data.

### Inference on real data

**Figure 4.**
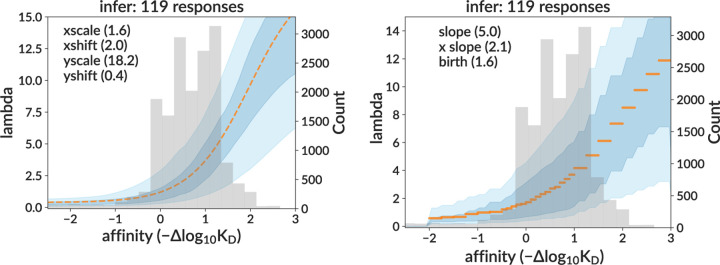
**Inferred response functions on real data** for sigmoid (left) and per-bin (right) models, corresponding to the non-sigmoid parameter values yielding simulation with the best-matching summary statistics. The medoid curve is shown in orange with 68% and 95% confidence intervals in blue, with observed affinity values in grey. Figure 4—figure supplement 1. Example inferred sigmoid curves on data for four representative GCs. Figure 4—figure supplement 2. Example inferred per-bin response functions on data for four representative GCs.

We then applied our neural network to real data. We show the inferred sigmoid response curves on the full sample for both the sigmoid and per-bin models (Figure 4), as well as several representative individual curve predictions (Figure 4—figure Supplement 1 and Figure 4—figure Supplement 2). The medoid inferred curve in orange is calculated by finding the curve that minimizes the difference to all other curves (see Methods).

**Figure 5.**
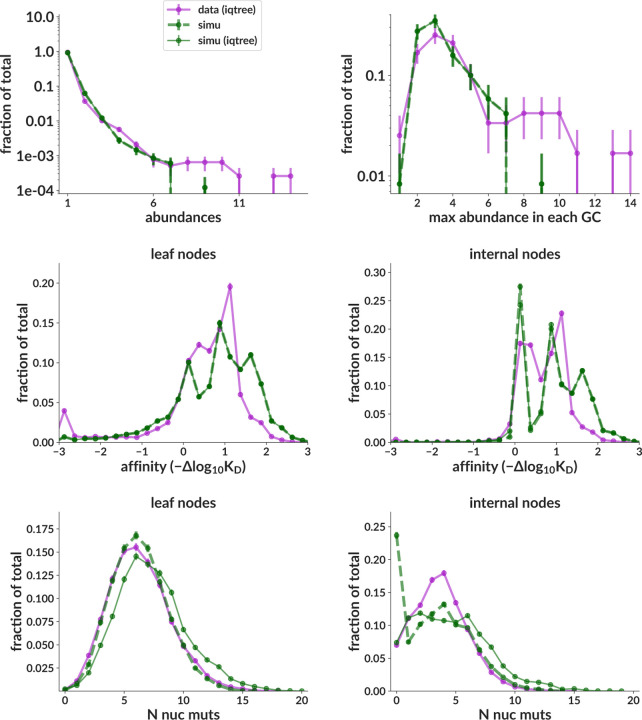
**Summary statistics on data vs simulation** for the central data mimic simulation sample that most closely mimics inferred data parameters. Simulation truth (dashed green) is unobservable and shown only for completeness; the important comparison is between purple and green solid lines, where both data and simulation have been run through IQ-TREE. Figure 5—figure supplement 1. Additional summary statistics distributions for the same central data mimic sample as the main figure. Figure 5—figure supplement 2. Summary statistic distributions for the simulation sample used for training.

As described above (see Methods), we run data inference many times, for many different values of three non-sigmoid parameters (carrying capacity, initial population, and death rate). This requires no retraining so is fast. This was implemented as a three-dimensional scan over four carrying capacity values (500, 750, 1000, 2000), three initial population values (8, 32, 128), and four death rates (0.05, 0.1, 0.2, 0.4). In Figure 4, we display only the inference corresponding to the values yielding simulation with the best summary statistics distributions: carrying capacity 500, initial population 128, and death rate 0.2. These summary statistics are shown in Figure 5 and Figure 5—figure Supplement 1. Discrepancies here are discussed in the Discussion.

**Figure 6.**
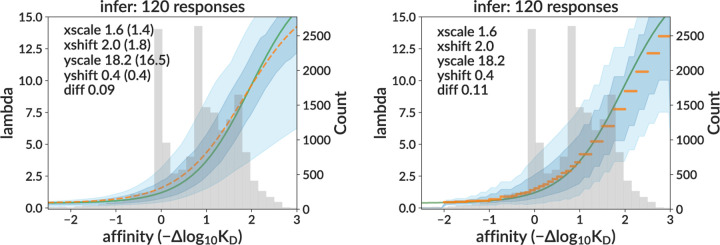
Inferred sigmoid curves for the central data mimic data-like simulation sample. The medoid curve is shown in orange, and true curve in green. This sample consists of 120 GCs all simulated with the same sigmoid parameters (from the central data prediction) and non-sigmoid parameters (from the best-matched summary statistics).

We then used simulation to explore expected performance on a sample that mimics our specific data. Specifically, we analyzed results on the central data mimic simulation, which uses the best-matching summary statistics from the non-sigmoid parameter scan (Figure 6). In contrast to the training sample, where each GC has different parameter values drawn from wide ranges, this sample consists of 120 GCs with identical parameters (except for N sampled sequences, see right column of Table 1). We found that the simulation and inference match each other well.

### Simulation mimics GC dynamics

It is important to establish how well our simulation mimics real BCR sequencing data. Since for the purposes of this paper we are interested in replay-style experiments, we seek to mimic these particular samples. In order to match data, we must use inferred values for both sigmoid and non-sigmoid parameters, using the procedure described above (Figure 5). The training sample, on the other hand, matches much less well because it is designed to span all plausible unknown parameter values to ensure that the neural network encounters any parameter combination that it is likely to see in data (Figure 5—figure Supplement 2). Note also that in practice our workflow involved testing inference on many data-like simulation samples with non-data-inferred parameters, before performing data inference.

### Effective birth rates

**Figure 7.**
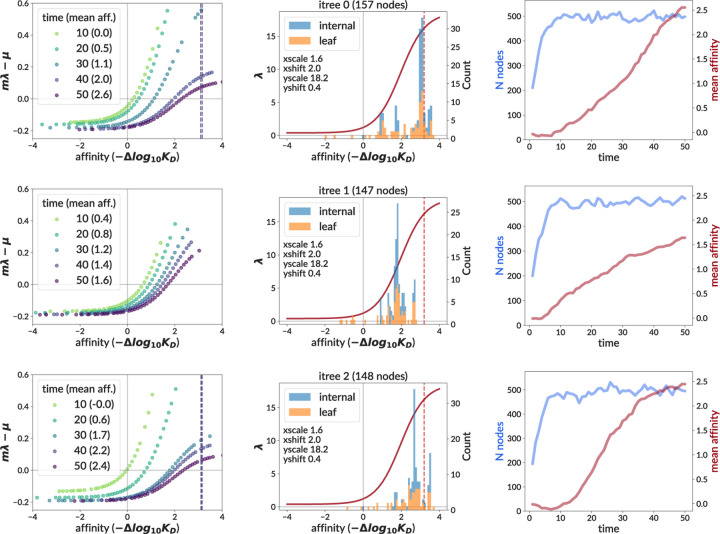
Effective birth rates (left column) calculated for three randomly-selected GCs from the central data mimic simulation sample. The effective birth rate for cell i is defined as $m\lambda_{i}-\mu_{i}$ for the carrying capacity modulating factor m, intrinsic birth rate $\lambda_{i}$, and death rate $\mu_{i}$. It is shown at several different time values for all living cells, except that for plotting clarity, cells closer to each other than 0.1 in affinity are not shown. Note that we extend time here to 50 days in order to aid comparison to the bulk data used by the traveling wave model (DeWitt et al., 2025, Fig. S6D), which assumes a steady state. (Our extracted GC data is sampled at 15 and 20 days.) Vertical dashed lines (left and middle columns) mark the point at which the slope has dropped to 1∕2 its maximum value (if absent, the slope never falls below this threshold). We also show the intrinsic birth rate λ (middle column, solid red curve), and include histograms of sampled affinities at the final time point. The right column shows time traces of the number of living cells (i.e. nodes, in blue) and the mean affinity of all living cells (red).

The birth rate described by the response function is an absolute quantity, λ, that determines the rate of cell division in the absence of carrying capacity constraints and cell death. However this is not the actual birth rate that is inverted to get a waiting time at each moment in the simulation. The simulation instead scales this rate by m (Eq 1) to maintain an upper limit to the population. It can thus be useful to calculate an effective birth rate $m\lambda_{i}-\mu_{i}$ describing the net fitness advantage of a cell i at a given moment in time. Here we have applied the carrying capacity modulation factor m (Eq 1) and subtracted the cell-specific death rate $\mu_{i}$. We now call the original λ the “intrinsic” birth rate to distinguish it from this effective rate. We show this effective birth rate on several GCs from the central data mimic simulation sample (Figure 7).

Overall, the effective rate has a much smaller scale, generally staying between −0.2 and 1 for most GCs, compared to 0 to more than 15 for the intrinsic rate. Note, however, that at small populations the effective rate would have the same range as the intrinsic rate, if we could plot it at the larger (unobserved) affinity values at which we can plot the intrinsic rate (see Discussion). The effective rate is also time-dependent, with m compressing its range as time goes on, which also contrasts with the fixed intrinsic rate. These changes continue well after the simulation has achieved maximum population size (blue line in right column, Figure 7). Biologically speaking, this means that there is less reward for gaining affinity later in the simulation than in the beginning. This feature is shared with the traveling wave model (DeWitt et al., 2025, Fig. S6D).

## Discussion

The germinal center reaction is a complex evolutionary process during which B cell populations improve their affinity. Little is known, however, about the specific form of the underlying relationship between affinity and fitness. Because a B cell’s fitness depends on the context of other cells with which it interacts, this relationship cannot be measured directly in a lab. Its form must instead be inferred based on observations of intact, evolving germinal centers.

We model this biology using a birth-death-mutation process with carrying capacity constraint. While birth-death models are common, the necessity of a capacity constraint makes likelihood evaluation intractable using existing methods (although recent theoretical work may provide a foundation for solving this in a “mean-field” sense as described in DeWitt et al., 2024). We thus pursued a likelihood-free approach, training a deep neural network on simulation designed to mimic the replay experiment. This likelihood-free approach also allows us to use a model of mutation on sequences that need not be Markov on affinity “types.”

We used this neural network to infer the affinity-fitness response function. Because the model includes several parameters that are not well-constrained by experiment, but whose prediction with the neural network would be infeasibly complicated, we adopted a two-step inference procedure. We first infer the sigmoid shape at many different potential non-sigmoid parameter values, then “fit” the non-sigmoid parameters using summary statistics. In testing on simulation, the network showed the ability to reliably infer the sigmoid’s shape on samples similar to the extracted GC data. When applied to that data, it inferred a consistent shape across trees that was in line with expectations from simulation, while also giving summary statistics well-matched to the data. This shape indicates that intrinsic fitness (defined as the rate of binary cell division without capacity constraint) roughly triples from affinity 0 (naive) to 1, and then triples again from 1 to 2. We did not observe any significant decrease in the response function slope (what we would call a ceiling on fitness) within the time range (20 days) of the GC extraction experiment, although we did see a decrease at affinities around 3.5, reached by day 50 in the data mimic simulation (Figure 7). This latter result should be treated with caution, however: this ceiling derives from an affinity region (see Figure 4) without observed affinities, and is thus essentially entirely reflective of our assumed sigmoid shape, rather than any direct information from data. We also note that the extracted GC data has very low levels of mutation compared to typical BCR repertoires, so a ceiling may manifest only at higher affinity values than we observe.

### Prior work

Measurements relevant to the affinity-fitness relationship

A variety of prior biological measurements help guide our expectations for the shape of the response function. The function’s slope, for instance, controls the speed of evolution in the GC, with a steeper slope creating stronger clonal bursts and faster selective sweeps. We are not, however, aware of any work numerically quantifying the shape of this important curve other than our recent manuscript (DeWitt et al., 2025). The existence and potential location of a ceiling on fitness, on the other hand, has been the subject of much work. Note that this is often referred to as an “affinity ceiling”, however because the proposed asymptote is flat on the vertical fitness (rather than horizontal affinity) axis, we instead refer to it as a “fitness ceiling”.

One study (Batista and Neuberger, 1998) measured how the B cell response to antigen varies with affinity, finding that association constant $K_{a}=1\text{/}K_{D}$ values above $10^{10}M^{-1}$ did not lead to increased B cell triggering. This value is roughly equal to one derived previously (Foote and Eisen, 1995) starting from two assumptions: that diffusion places a fundamental upper limit to $k_{\text{on}}$ (where $K_{D}=k_{\text{off}}\text{/}k_{\text{on}}$), and that dissociation half lives are less than one hour. Using our naive $K_{D}$ of $4x\;10^{-8}M$ (Tas et al., 2016), the ceiling $K_{a}$ value of $10^{10}M^{-1}$ corresponds to an affinity in our plots of $-log_{10}\frac{10^{-10}}{4x\;10^{-8}}=2.6$, a value at which we observe few enough values in data (Figure 4) that our inferred response function there is essentially meaningless. Also note that the additive, DMS-based affinity measurements that we use are only validated between about −1 and 2 (DeWitt et al., 2025, Fig. 2).

A minimum, “threshold” $K_{a}$ value of $10^{6}\;M^{-1}$, below which B cells do not respond, has also been reported (Batista and Neuberger, 1998). While we do not attempt to model such a threshold in our simulation, this value would correspond to an affinity of $-{\text{log}}_{10}\frac{10^{-6}}{4x\;10^{-8}}=-1.4$ in our plots, which is a region in which we observe the expected flat response function, although note that we also observe very few cells here (Figure 4).

### Inference of the affinity-fitness relationship

We have undertaken two other, independent efforts to infer the specific relationship between affinity and fitness, and it is instructive to compare their results. The approach in DeWitt et al. (2025) was to apply a traveling wave fitness approach (Neher, 2013; Nourmohammad et al., 2013) to the replay bulk data. This models the distribution of cell fitnesses $p\left(x,t\right)$ as a traveling wave, rather than, as we do here, treating the evolution of individual cells and their BCR sequences. It is based on the differential equation:

(8) $$\frac{\partial }{\partial t}p\left(x,t\right)=\underset{\text{Malthusian}\;\text{growth}}{\underset{︸}{\left(f\left(x\right)-\bar{f\left(t\right)}\right)p\left(x,t\right)}}+\underset{\text{net}\;\text{mutation}\;\text{to}\;\text{state}\;x}{\underset{︸}{\int_{-\infty }^{\infty }\left(q\left(y,x\right)p\left(y,t\right)-q\left(x,y\right)p\left(x,t\right)\right)dy,}}$$

which relates the time dependence of $p\left(x,t\right)$ to two quantities: the fitness landscape $f\left(x\right)$, and the mutational flux $q\left(x,y\right)$ between affinity states x and y, with $\bar{f\left(t\right)}$ the mean fitness of the population at time t. This fitness landscape $f\left(x\right)$ is an arbitrary function that is fit as part of the inference procedure. The coefficient $f\left(x\right)-\bar{f\left(t\right)}$ thus describes the net rate of population increase at time t for a cell with affinity x, and can be thought of as an effective birth rate and compared to our mλ−μ (Figure 7). We observe that our calculated mλ−μ values are of similar magnitude and shape to the f reported in DeWitt et al. (2025), although they are not identical.

This, however, is to be expected: although the models have similar intent, they have very different structures and assumptions and thus the outputs cannot be compared directly. For instance DeWitt et al. (2025) assumes a steady state with large (continuous) populations, whereas much of the dynamics of our model show effects of stochasticity from single cells or mutations. As time progresses in DeWitt et al. (2025) and mean population affinity increases, $f\left(x\right)-\bar{f\left(t\right)}$ shifts downwards without changing shape. In our model, on the other hand, the effective birth rate flattens and shifts rightwards with time as it is modulated by m and the mean population affinity increases (Figure 7). While both of these effects reflect the nature of selection in a dynamic population, their differences underline the fundamentally different viewpoints of the two models. Note also that, in order to facilitate comparison to DeWitt et al. (2025), the plots in Figure 7 extend to very small and very large affinity values (−4 to +4) about which we have essentially zero information from the extracted GC data (which extends from roughly −1 to +2). The effective birth rates in these outlying regions are thus almost entirely reflective of the prior assumptions of the models, rather than of information from the data. Finally, we note that the input for the traveling wave model in DeWitt et al. (2025) is count data from multiple time points, whereas data for our model are phylogenetic trees from a single time point.

Both the intrinsic and effective birth rates are useful quantities, with each being appropriate for different tasks. The intrinsic rate is an unchanging, fundamental feature of the GC system; but given a cell in a particular GC, it cannot tell us how many offspring to expect. The effective rate, on the other hand, tells us the expected number of offspring for each cell, but with the tradeoff that it has no fixed form: it depends, through m (Eq 1), on the sum over cells of both birth and death rates, as well as on the number of cells alive at that time. Also note that because it depends on the particular population of cells alive at a given time, it is not defined along the entire x axis, but only at x values at which living cells reside (calculating its value at an affinity that no cells have would presuppose adding a cell at that affinity, which would modify the calculation for all other cells).

We have also worked to infer the response function using likelihoods under a birth-death model (Bakis et al., 2025). This requires solutions of ordinary differential equations (ODEs) that account for unsampled lineages in birth-death models. Because these models require independent evolution of lineages, they are also not able to incorporate the effects of carrying capacity and competition between cells. We can nevertheless compare our effective birth rate to that inferred in Bakis et al. (2025). The main difference is the much earlier ceiling in Bakis et al. (2025), plateauing entirely by around x=0.5. This can be understood, however, as a consequence of the lack of population size constraint: without a carrying capacity, the model can only avoid unbounded population growth with an early, and low, fitness ceiling.

Recent work has developed birth-death processes with mean-field interactions (DeWitt et al., 2024) that could be used for inference under models with population size constraint. Although the theoretical promise for such models is clear, inference has not yet been implemented. In future work it would be interesting to compare these methods with those presented in this manuscript.

### Limitations

Our work is subject to a number of limitations, most importantly related to our model and simulation. As noted above, several aspects of our model’s organization may not accurately reflect real GC dynamics. Furthermore, even for aspects that are accurately modeled, chosen parameter values may either not reflect true biological values, or may represent subtly different “effective” parameters and thus also need modification. Our simulation also does not include recent results showing that the mutation rate may be depressed after a strong light zone signal (Pae et al., 2025a), or that lower affinity cells may be subject to higher SHM rates (Li et al., 2025).

Other possible misspecifications involve dark zone-light zone cycling and cell sampling. Our simulation implements a single-generation proliferative phase with mutation (dark zone) always followed by a single, separate selection phase (light zone). However, this may not accurately represent real GC dynamics; for instance this cannot simulate multiple proliferative phases between each selection phase (“clonal bursts”) (Tas et al., 2016; Pae et al., 2025b). It is also known that some selection occurs in the dark zone (Mayer et al., 2017), at least against nonfunctional cells, and our two-category death rate may not be sufficient to model this. We also sample uniform randomly from all live cells at the final time point, but it is possible that in real GCs cell sampling is based on more biased processes, for instance if higher affinity cells, or cells in a particular zone, are more likely to be sampled.

We also assume a single, constant death rate for functional cells. While we infer this rate with our summary statistic matching step, if the real rate depends on cell properties (other than simply being functional), the results will be incorrect. Because birth and death rates are interrelated mechanistically through phenomena such as tonic signaling (Long et al., 2022) and BCR expression-dependent birth rates (Lam et al., 1997), it is also unclear how much sensitivity we have to infer them independently. We note in passing that identifiability of birth and death rates from phylogenies is inherently difficult (Morlon et al., 2022).

It is also possible that our choice of a sigmoid for the response function is not sufficiently flexible. This form cannot, for instance, accommodate either a decrease in fitness with affinity, or a discontinuous change in slope. It also requires symmetry around its midpoint. Because both the sigmoid and per-bin models were trained on sigmoid simulation, they can only infer sigmoid-like response functions.

Our summary statistic distributions are not always well-matched to data. The worst discrepancies are inaccurate structure (extra peaks) in the simulation affinity distributions, and deficits in the tails of both simulation abundance plots. The extra affinity peaks could be from inaccuracies in the sequence-affinity mapping, which assumes zero epistasis. Enlarging the abundance tail is easily accomplished by increasing burstiness with a steeper response function, or by decreasing diversity via either smaller initial population or smaller carrying capacity. These changes also, of course, affect other distributions, with for instance a steeper response function increasing the speed of evolution and shifting the affinity distribution rightwards (while a smaller population does the opposite). While we can thus move the worst discrepancies from one distribution to another, our inability to match all distributions simultaneously is probably due either to one of the above model misspecifications, or to a sigmoid inference problem leading to incorrect response function.

Our neural network could also be improved. While the CBLV encoding incorporates a number of improvements over previous schemes, the process of encoding trees for digestion by neural networks is still a very new problem, and current methods are likely not particularly close to optimal. Recent work, for instance, has improved on the CBLV scheme by incorporating the generational context of each node in a way that makes it easier for the network to understand (Perez and Gascuel, 2024). This work also introduces a network architecture that is specifically designed around the new encoding. In the future, we plan to explore the use of this new method.

The replay experiment also represents a very special case among typical BCR repertoires. While the basic dynamics at play are likely to be representative of those in wild repertoires, it is important to keep in mind that these results only reflect mutation stemming from the exposure of a single naive antibody to a single experimental antigen. Some aspects of behavior in the low-SHMregime at shorter times of the extracted GC data are also potentially different to those at the higher SHM levels and longer times found in typical repertoires. Additionally, repeating the replay experiment using a less-optimized antigen with lower starting affinity would enable us to better map out dynamics during the early stages of affinity maturation when, for example, specificity may have some plasticity (Zuo et al., 2025).

### Conclusion and outlook

In closing, simulation-based methods have enabled inference on this otherwise-intractable, messy biological problem. However, they were not a panacea. Simulation-based inference is difficult when many parameters determine a model’s behavior, since the product of many dimensions cannot be tested exhaustively. The optimality surface was also found to be quite rugged: it was easy to find combinations of parameters that lead to pathologies. Previous successful studies using simulation-based inference on trees have had fewer parameters and a less complex objective (Voznica et al., 2022; Thompson et al., 2024). Neverthless, we were able to infer parameters of a complex and dynamic process that is hidden inside the germinal center.

Looking forward, even if by a miracle inference under an arbitrary forward-time model was available, interesting biological questions would remain. For instance, what mechanism limits the population size of the germinal center? In this paper we chose birth-limited population size control, and the validity of our results depends on this assumption. In comparison with the traveling wave model of DeWitt et al. (2025), which has separate assumptions, we could see that the inferred dynamics differ. Finding a convincing answer to this question will require additional experiments and likely additional modeling.

## Supplementary Material

Supplement 1

## Figures and Tables

**Table 1.** Simulation parameter values for training sample (left column, in which each GC has different parameters), and the central data mimic sample (right column, in which all GCs have the same, data-inferred parameters). Square brackets indicate a range, from which values are chosen either as detailed below (for sigmoid parameters) or uniformly at random.

	training	central data mimic
birth response function	sigmoid	sigmoid
xscale $\left(x_{c}\right)$	[0.01, 2]	1.6
xshift $\left(x_{h}\right)$	[−0.5, 3]	2.0
yscale $\left(y_{c}\right)$	[0.5, 35]	18.2
yshift $\left(y_{h}\right)$	[0, 0.6]	0.4
carrying capacity	[500, 2000]	500
capacity method	birth	birth
time to sampling	[10, 35]	20
# seqs/GC	[50, 130]	[60, 95]
# GCs	50,000	120
naive birth rate	[0.1, 15]	1.2
death rate (functional)	[0.05, 0.5]	0.2
death rate (stops)	10	10
initial population	[8–128]	128
mutability multiplier	0.68	0.5

**Table 2.** Neural network architecture.

order	type	filters	kernel size	pool size	stride length	N units	N params
1	1D convolutional	25	4				426
2	1D convolutional	25	4				2,525
3	max pooling			2	2		
4	1D convolutional	40	4				4,040
5	global avg. pooling						
6	dense					48	1,968
7	dense					32	1,568
8	dense					16	528
9	dense					8	136
10	dense					3	27

**Table 3.** Clip function bounds for neural network training.

parameter	min value	max value
xscale $\left(x_{c}\right)$	0.001	3.5
xshift $\left(x_{h}\right)$	−1.5	5
yscale $\left(y_{c}\right)$	0.1	65
yshift $\left(y_{h}\right)$	0	10
