# Supplementary material for: Inference of germinal center evolutionary dynamics via simulation-based deep learning
Source: ArXiv. 2025 Aug 13:arXiv:2508.09871v1. Preprint. [Version 1] (PMC12364052)
Supplement: Supplement 1 [file NIHPP2508.09871v1-supplement-1.pdf]

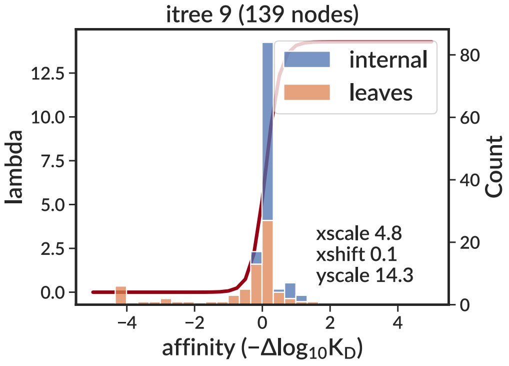

Figure 1—figure supplement 1. Example of simulation response function (red, left axis) and resulting node affinity values (histogram, right axis). The response function describes the relationship between affinity and fitness, while the node values show the actual affinity values of nodes in the resulting simulation for both internal (blue) and leaf (orange) nodes. Note that the count is not sampling the response function, so we do not expect the histogram and the function to match.

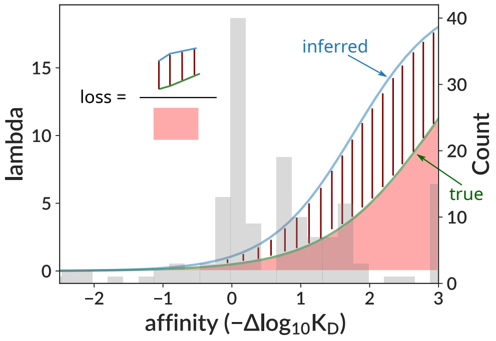

Figure 1—figure supplement 2. Curve difference loss function calculation. We divide the "difference" area (red bars) between the true (green) and inferred (red) curves by the area (light red) under the true curve within the bounds $[-2.5, 3]$.

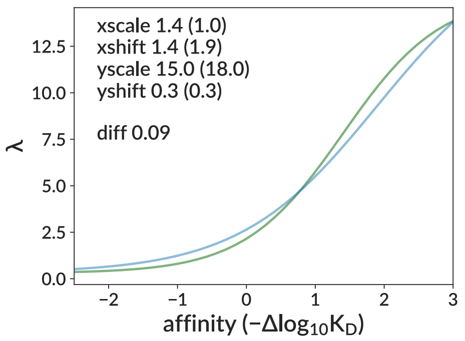

Figure 1—figure supplement 3. Example of approximate sigmoid parameter degeneracy. A potential pair of true (green) and inferred (blue) sigmoid curves, with true (inferred) parameter values. In the range shown, the inferred curve has compensated for a too-small `xscale` (transition steepness) by increasing `xshift` (shifting to the right) and `yscale` (upper asymptote). This results in a curve difference loss value of only 9%, which is much smaller our expected inference accuracy.

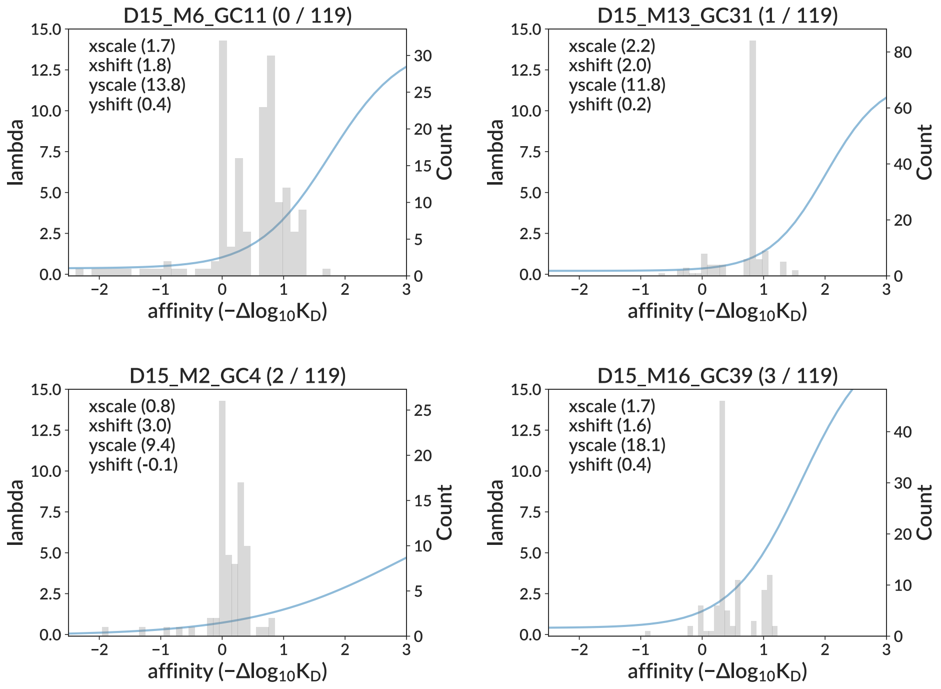

Figure 4—figure supplement 1. Example inferred sigmoid curves on data for four representative GCs.

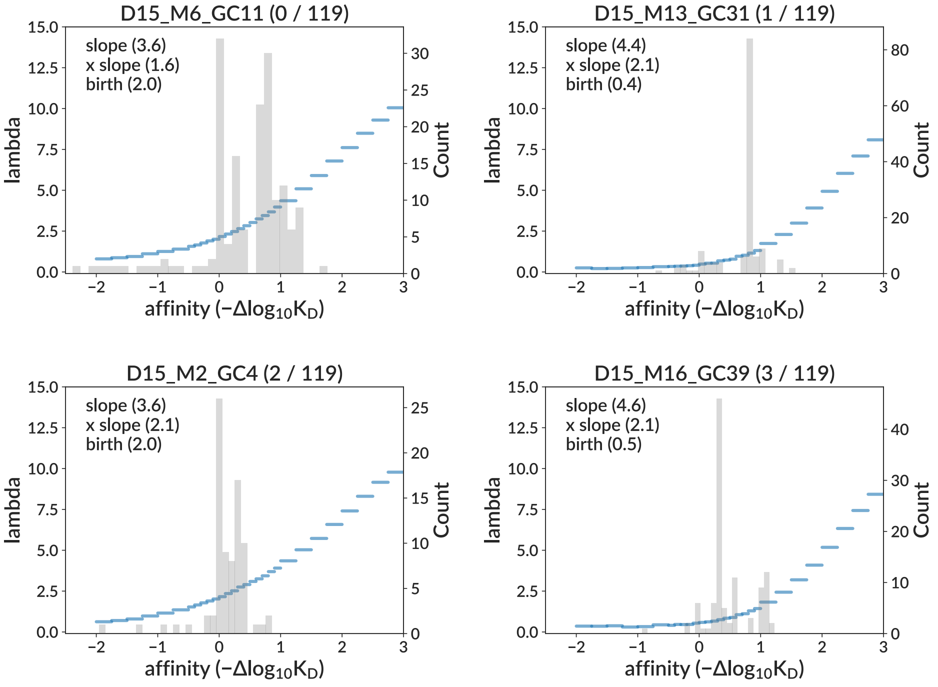

Figure 4—figure supplement 2. Example inferred per-bin response functions on data for four representative GCs.

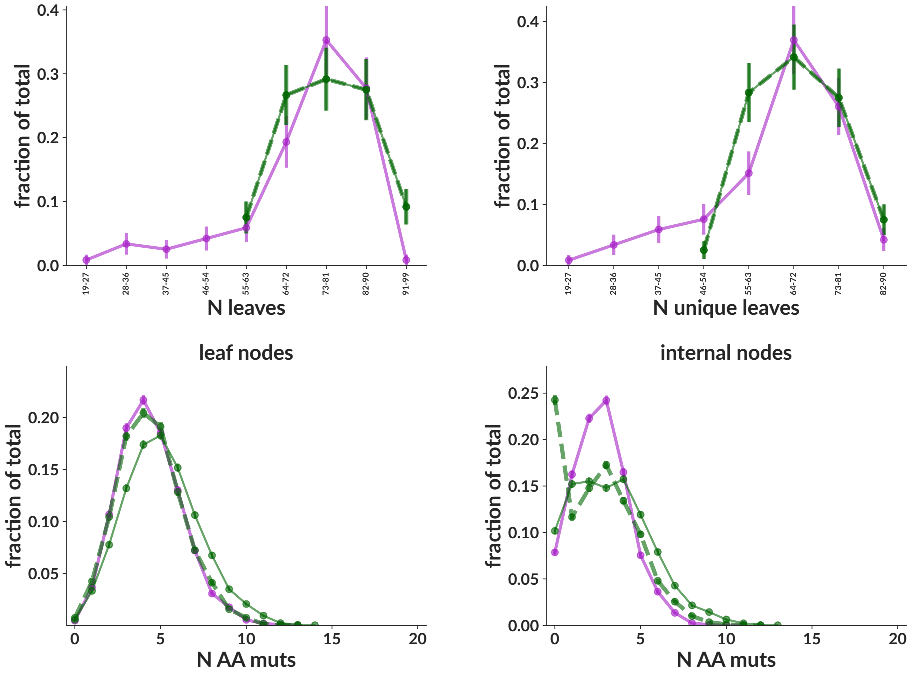

Figure 5—figure supplement 1. Additional summary statistics distributions for the same central data mimic sample as the main figure.

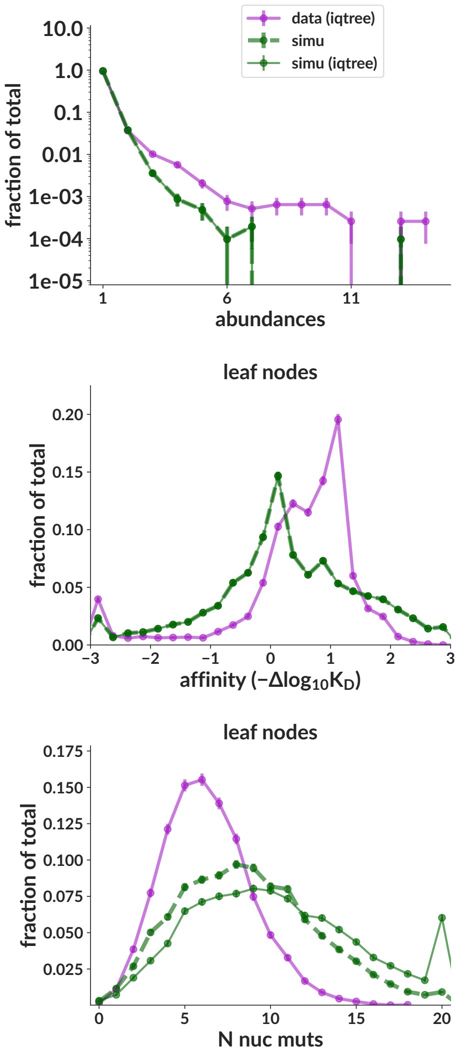

Figure 5—figure supplement 2. Summary statistic distributions for the simulation sample used for training. In order to allow easy comparison of abundance distributions, these plots include only 120 of the simulated trees. This sample is designed to have a wide range of parameters, encompassing all plausible true data values, and is thus not designed to closely match data summary statistics.
